# Clinical predictive factors of long‐term survival after curative resection of pancreatic cancer: a retrospective study

**DOI:** 10.1002/cam4.1178

**Published:** 2017-09-18

**Authors:** Yutaka Nakano, Minoru Kitago, Masahiro Shinoda, Yuta Abe, Hiroshi Yagi, Taizo Hibi, Ayano Takeuchi, Koichi Aiura, Osamu Itano, Yuko Kitagawa

**Affiliations:** ^1^ Department of Surgery Keio University School of Medicine Tokyo Japan; ^2^ Department of Preventive Medicine and Public Health Keio University School of Medicine Tokyo Japan; ^3^ Department of Surgery Kawasaki City Hospital Tokyo Japan; ^4^ Department of Gastrointestinal Surgery International University of Health and Welfare Chiba Japan

**Keywords:** Achieving 5‐year survival, neoadjuvant chemoradiotherapy, perioperative portal vein infusion chemotherapy, predictive factors, preoperative serum albumin

## Abstract

Pancreatic ductal adenocarcinoma (PDAC) continues to have the poorest prognosis of all gastrointestinal malignancies, even after the tumor has been completely resected. However, only a proportion of patients achieve 5‐year survival after resection. The factors predictive of achieving 5‐year survival remain unclear. The aim of this study was to investigate the pre‐ and postoperative clinicopathological characteristics of PDAC patients with a >5‐year survival after curative resection. We retrospectively reviewed patients who underwent pancreatectomy for PDAC between January 1995 and December 2011. Logistic regression analysis was performed to determine the predictive factors for 5‐year survival. One hundred and fifty‐one patients were enrolled, including 38 patients with 5‐year survival (actual 5‐year survival rate, 25.2%). The independent preoperative factors predictive of achieving 5‐year survival included serum albumin levels (odds ratio [OR]: 5.06, 95.0% confidence interval [CI]: 1.49–17.19; *P *=* *0.009) and neoadjuvant chemoradiotherapy (OR: 3.02, 95.0% CI: 1.00–9.08; *P *=* *0.049). Venous infiltration (OR: 2.99, 95.0% CI: 1.09–8.25; *P *=* *0.034), liver recurrence (OR: 0.17, 95.0% CI: 0.04–0.69; *P *=* *0.013), and perioperative portal vein infusion chemotherapy (OR: 3.06, 95.0% CI: 1.09–8.25; *P *=* *0.028) were found to be independent postoperative predictive factors for achieving 5‐year survival. Serum albumin levels could be a biomarker for predicting the prognosis of PDAC patients after curative resection. Liver recurrence and perioperative portal vein infusion chemotherapy were independent postoperative factors, suggesting that perioperative portal vein infusion chemotherapy could be promising for improving the survival rate of PDAC patients after curative resection.

## Introduction

The 5‐year survival rate of pancreatic ductal adenocarcinoma (PDAC) patients remains poor relative to other gastrointestinal malignancies 1, 2, even after complete curative resection. Recently, the 5‐year survival rates have improved due to various developments in pre‐ and postoperative chemo‐ and chemoradiotherapy. Neoadjuvant chemoradiotherapy (NACRT) is considered an acceptable treatment for locally advanced PDAC 3, 4, and postoperative adjuvant chemotherapy has been widely administered 5, 6. However, the 5‐year survival rate of PDAC patients remains low despite the development of these multimodal therapies.

Several retrospective studies 7, 8, 9 have reported that a proportion of patients who underwent curative resection for PDAC have survived for >5 years. Almost all the factors predictive of 5‐year survival in these studies 7, 8, 9 are associated with pathological data (e.g., resection margin and lymph node metastasis status). However, clinical factors that predict long‐term survival in PDAC patients, especially pre‐ and postoperative chemo‐ or chemoradiotherapy and preoperative laboratory data, have yet to be fully elucidated.

In this study, we aimed to investigate pre‐ and postoperative clinicopathological characteristics of PDAC patients who survived for >5 years after curative resection, focusing on pre‐ and postoperative chemo‐ or chemoradiotherapy and preoperative laboratory data.

## Materials and Methods

### Patients

Patients who underwent intended curative pancreatectomy for PDAC at our institution between January 1995 and December 2011 were retrospectively reviewed. All patients were histologically confirmed as having PDAC. We excluded patients who underwent R2 resections. All study participants had provided informed written consent. The Human Experimentation Committee of our institution approved this study. Research was conducted in accordance with the Declaration of Helsinki.

Patients were stratified into two groups according to survival: the 5‐year survival group (patients who survived for >5 years) and the <5‐year survival group (patients who died or were lost to follow‐up within 5 years of curative pancreatectomy).

### Preoperative assessment

The demographic and clinical variables included age, gender, body mass index, operative procedure, NACRT, tumor size, and preoperative laboratory data. The biochemical profile included total bilirubin, albumin, and globulin levels; the albumin‐to‐globulin ratio; 10 cholinesterase and total cholesterol levels; white blood cell [WBC] count; neutrophil, lymphocyte, and platelet counts; the neutrophil‐to‐lymphocyte ratio 11. Markers such as C‐reactive protein [CRP], fibrinogen, carcinoembryonic antigen [CEA], carbohydrate antigen 19‐9 [CA 19‐9], elastase‐1, duke pancreatic monoclonal antigen type‐2 [DUPAN‐2], and s‐pancreas antigen‐1 [SPAN‐1] levels; and the CRP‐to‐albumin ratio 12 were included. The prognostic nutritional index (PNI) 13 and modified Glasgow Prognostic Score (mGPS) 14, useful predictors of prognosis in PDAC patients, were also included.

Since 2003, at our institution, NACRT has been administered to patients who were diagnosed with T3‐4 disease according to the Union for International Cancer Control (UICC) TNM Classification of Malignant Tumors (seventh edition). NACRT consisted of 40.0 Gy of radiation (2.0 Gy/day, days 1–5 each week, for 4 weeks), 5‐fluorouracil (5‐FU; 300.0 mg/day by continuous intravenous infusion, days 1–5 each week, for 4 weeks), mitomycin C (4.0 mg/[body∙day] by intravenous bolus, days 1, 8, 15, and 22), cisplatin (10.0 mg/[body∙day] by intravenous bolus, days 2, 9, 16, and 23), and heparin (6000 IU/[body∙day] by continuous intravenous infusion, days 1–7 each week for 4 weeks) 15.

### Surgical resection and pathology

Surgical resection included pancreaticoduodenectomy, distal pancreatectomy, and total pancreatectomy. D2 lymph node dissections were performed in all patients.

Pathological staging of all specimens was determined according to the UICC TNM Classification of Malignant Tumors (seventh edition). R0 resections were defined as cases without gross or microscopic evidence of residue disease. R1 resections had microscopically positive margins and R2 resections still contained some gross tumor matter. Pathological features associated with prognosis included histologically assessed tumor size; distal bile duct, duodenal, serosal, retropancreatic tissue, portal venous or arterial system, and extrapancreatic nerve plexus invasion; other organ invasion; lymph node metastasis; and lymphatic, venous, or intrapancreatic neural infiltration 16.

### Postoperative assessment

Since the year 1986, perioperative portal vein infusion (PVI) chemotherapy has been performed as a standard treatment to prevent liver recurrence and improve survival in pancreatic cancer patients undergoing potentially curative resection at our institution. PVI was adopted in only those patients who gave informed consent for this treatment. PVI chemotherapy consisted of 5‐FU and heparin (250.0 mg/day and 2000 IU/[body∙day], days 1–7 each week for 4 weeks), mitomycin C (4.0 mg/[body∙day], days 6, 13, 20, and 27), and cisplatin (10.0 mg/[body∙day], days 7, 14, 21, and 28) 17, 18. S‐1, gemcitabine, 5‐FU, or mitomycin C was administered as adjuvant chemotherapy at the physician's discretion. Postoperative complications were evaluated according to the Clavien‐Dindo classification. In this study, pancreatic fistulas, intra‐abdominal bleeding, delayed gastric emptying, and fluid collection were also investigated.

### Follow‐up

Patients were followed‐up 1, 3, 6, and 12 months after surgery. Patients were also subject to semiannual reviews. Clinical examinations, laboratory investigations, and abdominal computed tomography scans (to detect tumor recurrence) were performed. Disease‐free survival (DFS) was calculated from the date of surgery to the date of recurrence or last follow‐up and overall survival (OS) was calculated from the date of surgery to the date of death or last follow‐up. Patients were followed up until death or December 2016.

### Statistical analyses

The clinicopathological characteristics between the 5‐year survival and <5‐year survival groups were evaluated and factors predictive of achieving 5‐year survival were investigated using logistic regression analysis. The optimal cut‐off points for predicting 5‐year survival were estimated from a receiver operating characteristic (ROC) curve analysis. Survival curves were plotted using the Kaplan–Meier method and compared by the log‐rank test. Variables that were significant in the univariate analysis were included in the multivariate analysis to identify prognostic factors of survival. All statistical analyses were conducted using Statistical Package for the Social Sciences for Macintosh, software version 23.0 (IBM Corp., Armonk, NY). A *P *<* *0.05 was considered statistically significant.

## Results

### Patient characteristics and surgical procedures

In total, 153 patients underwent curative resection for PDAC between January 1995 and December 2011. Two patients who underwent R2 resections were excluded. Therefore, 151 patients were enrolled in the final study. The patient characteristics are summarized in Table 1.

**Table 1:** Patient characteristics

Characteristic	Patients (*N* = 151)
Gender, *N* (%)	
M	94 (62.3)
F	57 (37.7)
Age (years), median (range)	67 (46–83)
NACRT, *N* (%)	23 (15.2)
Surgical procedure, *N* (%)	
Pancreaticoduodenectomy	96 (63.6)
Distal pancreatectomy	48 (31.8)
Total pancreatectomy	7 (4.6)
UICC Stage, *N* (%)^a^	
IA	6 (4.0)
IB	7 (4.6)
IIA	36 (23.8)
IIB	89 (58.9)
III	1 (0.7)
IV	12 (8.0)
Perioperative PVI chemotherapy, *N* (%)	79 (52.3)
Adjuvant chemotherapy, *N* (%)^b^	43 (28.5)
LNM, *N* (%)	
Positive	101 (66.9)
Negative	50 (33.1)
Resection status, *N* (%)	
R0	110 (72.8)
R1	41 (27.2)

5‐FU, 5‐fluorouracil; F, female; LNM, lymph node metastasis; M, male; NACRT, neoadjuvant chemoradiotherapy; PVI, portal vein infusion; UICC, Union for International Cancer Control.

a Pathological stage, UICC TNM Classification of Malignant Tumors (seventh edition).

b S‐1, gemcitabine, mitomycin C, or 5‐FU.

Of the 151 patients, 38 (25.2%) were included in the 5‐year survival group and 113 (74.8%) were included in the <5‐year survival group. The actual 5‐year survival rate was 25.2%. The median follow‐up duration of the 5‐year survival group was 84 (range, 60–197) months. Sixteen patients (42.1%) survived without recurrence and seven patients (18.4%) survived with recurrence (local [*N* = 3], lungs [*N* = 2], liver [*N* = 1], and bone [*N* = 1]). Eleven patients (28.9%) died of PDAC and four patients (10.5%) died of other causes. The patterns of initial recurrence were local (*N* = 9; 23.7%), followed by the lungs (*N* = 6; 15.8%), liver (*N* = 4; 10.5%), lymph nodes (*N* = 4; 10.5%), and bone (*N* = 1; 2.6%).

### Pre‐ and postoperative outcomes in the 5‐year survival and <5‐year survival groups

The preoperative data are summarized in Table 2. Several factors were identified in the univariate analysis as being significantly different between the 5‐year survival and <5‐year survival groups, including NACRT, serum albumin levels, and the WBC and lymphocyte counts.

**Table 2:** Univariate analysis of preoperative predictive factors

Factor	5‐year survival (*N* = 38)	<5‐year survival (*N* = 133)	*P*‐value
Age (years), median (range)	65 (55–81)	67 (46–83)	0.211
Sex (M/F)	24/14	70/43	0.894
BMI (kg/m^2^), median (range)	23.8 (16.4–40.0)	23.9 (15.8–33.4)	0.650
Surgical procedure, *N* (%)			0.226
Pancreaticoduodenectomy	20 (52.6)	76 (67.3)	0.121
Distal pancreatectomy	15 (39.5)	33 (29.2)	0.314
Total pancreatectomy	3 (7.9)	4 (3.5)	0.369
NACRT, *N* (%)	12 (31.5)	11 (9.7)	0.003*
Tumor size (cm), *N* (%)			
<4.0	35.0 (92.1)	101.0 (89.4)	0.628
≥4.0	3.0 (7.9)	12.0 (10.6)	0.628
Total bilirubin level (mg/dL), median (range)	0.8 (0.3–4.2)	0.9 (0.4–6.5)	0.252
Albumin level (g/L), median (range)	4.0 (2.6–4.7)	3.8 (2.7–4.8)	0.011*
Globulin level (g/L), median (range)	2.9 (0.3–4.0)	2.9 (1.9–4.2)	0.981
Albumin‐to‐globulin ratio, median (range)	0.3 (0.1–1.7)	0.3 (0.1–2.5)	0.129
Cholinesterase level (U/L), median (range)	248.0 (76.0–394.0)	241.0 (72.0–427.0)	0.664
Total cholesterol level (mg/dL), median (range)	164.0 (94.0–257.0)	176.0 (94.0–303.0)	0.658
WBC count (×10^3^/*μ*L), median (range)	4550 (1400–8400)	5300 (2000–12,400)	0.014*
Neutrophil count (×10^3^/*μ*L), median (range)	2881 (1106–7039)	3088 (1016–9696)	0.057
Lymphocyte count (×10^3^/*μ*L), median (range)	1093 (164–2144)	1312 (453–3248)	0.019*
Neutrophil‐to‐lymphocyte ratio, median (range)	2.3 (1.0–7.4)	2.3 (0.5–11.0)	0.584
Platelet count (×10^3^/*μ*L), median (range)	197 (93–410)	216 (91–411)	0.085
CRP level (mg/L), median (range)	0.2 (<0.1–9.6)	0.2 (<0.1–5.2)	0.545
Fibrinogen level (mg/dL), median (range)	330.0 (130.0–714.0)	355.0 (182.0–669.0)	0.190
CEA level (ng/mL), median (range)	2.6 (0.8–52.6)	3.0 (0.9–60.9)	0.425
CA 19‐9 level (U/mL), median (range)	45.0 (1.0–7240.0)	88.0 (1.0–8820.0)	0.814
Elastase‐1 level (ng/dL), median (range)	101.0 (40.0–1431.0)	215.0 (31.0–5230.0)	0.112
DUPAN‐2 level (U/mL), median (range)	42.0 (25.0–3930.0)	175.0 (25.0–18,400.0)	0.186
SPAN‐1 level (U/mL), median (range)	25.0 (6.0–228.0)	71.0 (6.0–3100.0)	0.091
PNI, median (range)	45.4 (34.8–54.6)	45.4 (32.2–62.3)	0.214
CRP‐to‐albumin ratio, median (range)	0.040 (0.010–2.230)	0.040 (0.002–1.410)	0.724
mGPS (0/1/2), *N*	31/7/0	78/30/5	0.080

BMI, body mass index; CA 19‐9, carbohydrate antigen 19‐9; CEA, carcinoembryonic antigen; CRP, C‐reactive protein; DUPAN‐2, Duke pancreatic monoclonal antigen type‐2; F, female; M, male; mGPS, modified Glasgow Prognostic Score; NACRT, neoadjuvant chemoradiotherapy; PNI, prognostic nutritional index; SPAN‐1, s‐pancreas antigen‐1; WBC, white blood cell.

*P *<* *0.05.

From the multivariate analysis, NACRT (odds ratio [OR]: 3.02, 95.0% confidence interval [CI]: 1.00–9.08; *P *=* *0.049) and serum albumin levels (OR: 5.06, 95.0% CI: 1.49–17.19; *P *=* *0.009) were confirmed as being independent predictive factors for achieving 5‐year survival (Table 3).

**Table 3:** Multivariate analysis of preoperative predictive factors for achieving 5‐year survival

Factor	*P*‐value	OR	95.0% CI
NACRT	0.049*	3.02	1.00–9.08
Albumin level (g/L)	0.009*	5.06	1.49–17.19
WBC count (×10^3^/*μ*L)	0.205	1.00	1.00–1.00
Lymphocyte count (×10^3^/*μ*L)	0.432	1.00	0.99–1.00

CI, confidence interval; NACRT, neoadjuvant chemoradiotherapy; OR, odds ratio; WBC, white blood cell.

a *P *<* *0.05.

The postoperative outcomes in these groups are displayed in Table 4. Lymphatic, venous, and neural infiltration; lymph node metastasis; pathological Stage IA and IIA‐B; perioperative PVI chemotherapy; and local, liver, and peritoneal recurrences were identified as predictive factors for achieving 5‐year survival in the univariate analysis. These factors were further analyzed in the multivariate analysis (Table 5). Venous infiltration (0–1) (OR: 2.99, 95.0% CI: 1.09–8.25; *P *=* *0.034), perioperative PVI chemotherapy (OR: 3.06, 95.0% CI: 1.09–8.25; *P *=* *0.028), and liver recurrence (OR: 0.17, 95.0% CI: 0.04–0.69; *P *=* *0.013) were confirmed as independent and significant predictive factors for achieving 5‐year survival.

**Table 4:** Univariate analysis of postoperative predictive factors

Factor	5‐Year survival (*N* = 38)	<5‐Year survival (*N* = 113)	*P*‐value
Operative time (minutes), median (range)	599 (259–1032)	587 (187–1173)	0.934
Blood loss (g), median (range)	583.0 (50.0–2800.0)	788.0 (30.0–5662.0)	0.278
Postoperative complication, *N* (%)			
Pancreatic fistula	7 (18.4)	36 (31.9)	0.117
Intra‐abdominal bleeding	1 (2.6)	7 (6.2)	0.411
Delayed gastric emptying	1 (2.6)	7 (6.2)	0.411
Fluid collection	5 (13.2)	12 (10.6)	0.669
Lymphatic infiltration, *N* (%)			
0–1	27 (71.1)	54 (47.8)	
2–3	11 (28.9)	59 (52.2)	0.015^a^
Venous infiltration, *N* (%)			
0–1	25 (65.8)	43 (38.1)	
2–3	13 (34.2)	70 (61.9)	0.004^a^
Neural infiltration, *N* (%)			
0–1	21 (55.3)	33 (29.2)	
2–3	17 (44.7)	80 (70.8)	0.005^a^
LNM, *N* (%)			
Positive	16 (42.1)	87 (77.0)	
Negative	22 (57.9)	26 (23.0)	<0.001^a^
Resection status, *N* (%)			
R0	32 (84.2)	78 (69.0)	0.074
R1	6 (15.8)	35 (31.0)	0.108
UICC stage, *N* (%)^b^			
IA	5 (13.2)	1 (0.9)	0.011^a^
IB	3 (7.9)	4 (3.5)	0.282
IIA	14 (36.8)	22 (19.5)	0.032^a^
IIB	14 (36.8)	75 (66.4)	0.002^a^
III	0 (0.0)	3 (2.7)	1.000
IV	2 (5.3)	10 (8.8)	0.484
Perioperative PVI chemotherapy, *N* (%)	27 (71.1)	52 (46.0)	0.009^a^
Adjuvant chemotherapy, *N* (%)^c^	13 (34.2)	30 (26.5)	0.367
Recurrence, *N* (%)			
Local	9 (23.7)	59 (52.2)	0.003^a^
Liver	4 (10.5)	45 (39.8)	0.002^a^
Lung	6 (15.8)	12 (10.6)	0.398
Peritoneal	4 (10.5)	44 (38.9)	0.003^a^

5‐FU, 5‐fluorouracil; LNM, lymph node metastasis; PVI, portal vein infusion; UICC, Union for International Cancer Control.

a *P *<* *0.05.

b Pathological stage, UICC Classification of Malignant Tumors (seventh edition).

c S‐1, gemcitabine, mitomycin C, or 5‐FU.

**Table 5:** Multivariate analysis of postoperative predictive factors for achieving 5‐year survival

Factor	*P*‐value	OR	95.0% CI
Infiltration (0–1)			
Lymphatic	0.458	0.64	0.20–20.70
Venous	0.034^a^	2.99	1.09–8.25
Neural	0.160	2.10	0.75–5.95
LNM (+)	0.947	0.92	0.07–12.52
UICC stage^b^			
IA	0.116	12.33	0.54–283.60
IIA	0.475	2.07	0.28–15.35
IIB	0.474	0.46	0.28–15.35
Perioperative PVI chemotherapy	0.028^a^	3.06	1.09–8.25
Recurrence			
Local	0.257	0.51	0.16–1.63
Liver	0.013^a^	0.17	0.04–0.69
Peritoneal	0.076	0.28	0.07–1.15

CI, confidence interval; LNM, lymph node metastasis; OR, odds ratio; PVI, portal vein infusion; UICC, Union for International Cancer Control.

a *P *<* *0.05.

b Pathological stage, UICC Classification of Malignant Tumors (seventh edition).

### Preoperative albumin levels and survival

**Figure 1:**
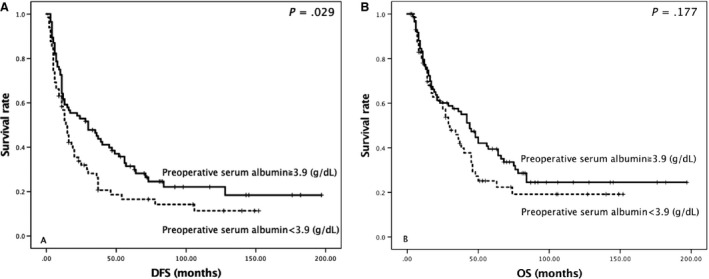
Kaplan–Meier curves of (A) postoperative disease‐free survival (DFS) and (B) overall survival (OS) according to preoperative serum albumin levels.

ROC curve analysis demonstrated that a preoperative serum albumin level of 3.9 g/dL (area under the curve, 0.670; *P *=* *0.002) was the optimal cut‐off for 5‐year survival. In the survival analysis according to the Kaplan–Meier method, patients with preoperative serum albumin levels of ≥3.9 g/dL had a significantly longer DFS compared to patients with preoperative serum albumin levels of <3.9 g/dL (Fig. 1A; *P *=* *0.029). The OS of these patients was also longer, although this was not significant (Fig. 1B).

### Other predictive factors and survival

**Figure 2:**
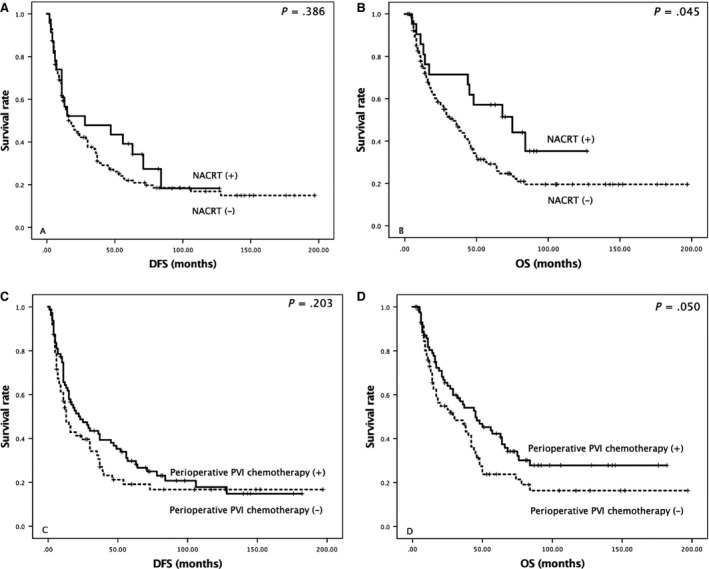
Kaplan–Meier curves of (A, C) postoperative disease‐free survival (DFS) and (B, D) overall survival (OS) according to neoadjuvant chemoradiotherapy (NACRT) and perioperative adjuvant portal vein infusion (PVI).

Patients receiving NACRT did not have a significantly longer DFS (Fig. 2A). However, they did have a significantly longer OS (Fig. 2B; *P *=* *0.045). Adjuvant PVI chemotherapy was not a significant predictor of DFS (Fig. 2C), but was predictive of OS (Fig. 2D; *P *=* *0.05).

## Discussion

This study investigated the pre‐ and postoperative predictive factors related to survival in patients with 5‐year survival compared to those with <5‐year survival. Our findings demonstrated that NACRT and preoperative serum albumin levels were independent preoperative predictive factors for achieving 5‐year survival. Additionally, venous infiltration, perioperative PVI chemotherapy, and liver recurrence were confirmed as independent postoperative predictive factors for achieving 5‐year survival.

By analyzing patients who survived for >5 years, factors for achieving 5‐year survival could be identified more accurately. Previous retrospective studies 7, 8, 9 have identified preoperative serum CA 19‐9 levels, multiple operations, lymph node metastasis, and R0 resections as prognostic factors in PDAC patients with an actual 5‐year survival after curative resection. These studies 7, 8, 9 also reported 5‐year survival rates of 10.2–27.0%, although the periods of study varied. In this study, we reveal novel predictive factors: preoperative serum albumin levels, NACRT, perioperative PVI chemotherapy, venous infiltration, and liver recurrence.

NACRT has been used to reduce tumor volume and improve margin‐negative resection rates in patients with locally advanced PDAC 4, 19. A clinical trial 20 has made progress. However, standardized protocols have yet to be established. At our institution, we began administering NACRT in 2003. We have previously reported 19 on two cases of PDAC with pathological complete responses to NACRT and have included these cases in our 5‐year survival group. Katz et al. 9 emphasized the importance of staging and patient selection, a standardized operative approach, and the routine use of multimodal therapy for achieving 5‐year survival rates.

Previous studies 21, 22 have suggested that in patients with non–small‐cell lung cancer or epithelial ovarian cancer, preoperative serum albumin levels are associated with a predictive survival rate. Low serum albumin levels are associated with a risk of postoperative complications and poor survival outcomes because of malnutrition 23. The prognostic factors related to serum albumin levels (e.g., CRP‐to‐albumin ratio, PNI, and mGPS) 12, 13, 14 have previously been identified in PDAC patients. However, few studies have reported that preoperative serum albumin levels alone can predict survival. Using prospective data from two randomized controlled trials 24, 25, Stocken et al. 26 have identified serum albumin as one of the prognostic factors for survival in PDAC. We believe that preoperative serum albumin levels may represent a useful biomarker for predicting the prognosis of PDAC patients after resection.

We hypothesize that preoperative albumin levels may be influenced by several factors, including systemic inflammation, disease state, nutritional status, liver cirrhosis, and the quantity of skeletal muscle 27, 28, 29, 30, 31. Several studies 32, 33, 34 have demonstrated that preoperative inflammation and postoperative complications can lead to poor survival outcomes in patients with various types of tumors. In our study, preoperative CRP levels, WBC counts, and fibrinogen levels, measured acute or chronic inflammatory markers, did not correlate with preoperative albumin levels (data not shown). Therefore, we speculate that preoperative serum albumin levels may reflect nutritional depletion and loss of skeletal muscle.

It remains unclear whether improving preoperative serum albumin levels can lead to a favorable prognosis. The criteria of the European Society for Clinical Nutrition and Metabolism 35 demonstrate that low serum albumin levels is an established marker of malnutrition, and improving perioperative nutrition, especially immunonutrition, can result in reduced postoperative complications and hospital stays. In pancreatectomy, preoperative providing oral immunonutrition helps in reducing risk of postoperative complications such as infections, and also the length of hospital stays 36. From a molecular viewpoint, the overexpression of caspases and the reduced levels of antiapoptotic proteins in PDAC patients may lead to lymphocyte apoptosis and dysfunction and immune system suppression, especially after pancreatectomy 37. Therefore, improving perioperative immunonutrition may reduce the risk of immune disorders in patients with PDAC.

We consider it important to control for disease state and cancer recurrence. Among the 5‐year survivors, recurrence (especially liver recurrence) was a poor prognostic factor. To prevent liver recurrence and improve survival in PDAC patients who undergo potentially curative resections, we have administered perioperative PVI chemotherapy with 5‐FU at our institution since 1986 17. We have already reported that perioperative PVI chemotherapy is beneficial in preventing liver recurrence 18, and in this study, perioperative PVI chemotherapy was identified as an independent postoperative predictive factor for achieving 5‐year survival.

In our univariate analysis, the pattern of initial recurrence (except lung recurrence) was a significant risk factor for achieving 5‐year survival. Several studies 38, 39, 40 have revealed that patients with isolated lung recurrence as the initial recurrence followed a better course than patients with other recurrences 38 for a variety of reasons. First, lung recurrence was more likely to occur late with a longer time to development than other sites of recurrence 39. Second, lung recurrence may be associated with unique and different phenotypes to those with other patterns of lung metastases in PDAC patients 38. Finally, the outcomes of patients who underwent reoperation for isolated lung recurrence were superior 40. In this study, patients with lung recurrence as the initial recurrence survived longer than patients with other patterns of recurrence (median OS: 45 vs. 18 months; *P *=* *0.008).

There are several limitations of this study. First, this was a retrospective study conducted at a single institution. Second, it was unclear whether improving preoperative serum albumin levels resulted in long‐term survival benefits after resection. Although the relationship between improving serum albumin levels and variations in oncological behavior is not well understood, several studies 26, 28, 29, 41 have demonstrated an association between hypoalbuminemia and poor postoperative short‐/long‐term survival in patients with various types of malignancies. We believe that preoperative serum albumin levels represent a potential biomarker for predicting prognosis in PDAC patients after curative resection. However, further studies will need to be conducted in order to evaluate the relationship between serum albumin levels and improved survival after pancreatectomy.

In conclusion, preoperative serum albumin levels may represent a useful biomarker for predicting prognosis in PDAC patients after curative resection. This factor was more straightforward in clinical situations than other prognostic factors that are related to serum albumin levels (e.g., the CRP to albumin ratio, PNI, and mGPS). NACRT and perioperative PVI chemotherapy were also identified as significant predictive factors. Patients with liver recurrence were more likely to have a poor prognosis. Therefore, perioperative chemotherapy (e.g., PVI) is well suited to improve survival after pancreatectomy.

## Conflict of Interest

Yuko Kitagawa has received research funding from Kyowa Hakko Kirin Co., Ltd., Yakult Honsha Co., Ltd., Eli Lilly Japan K.K., and Taiho Pharmaceutical Co., Ltd. The remaining authors have no conflicts of interest to disclose.
